# Removal of Cystic Tumors from the Eyelids

**Published:** 1873-06

**Authors:** 


*Removal of Cystic Tumors from the Eyelids.*—Professor J. J. Chisholm, M. D., of Baltimore, gives the following simple method of removing these tumors, which is published in the *Canada Medical Journal:*

"It is a modification in the use of the nitrate of silver that Dr. Chisholm has found so effective in the treatment of sebaceous cysts of the lid, and which has enabled him to discard for many years, the tedious, painful, and sometimes dangerous cutting out of such tumors. If the tumor be a sebaceous cyst, located between the upper portion of the tarsal cartilage and the skin, a Dessmarre's ring forceps is used as a clamp upon the lid, to shield the ball of the eye from injury, to fix the tumor, and to prevent annoying oozing of the blood. Under this ring pressure a small opening is made into the cyst, through which its contents are squeezed out. The end of a small silver probe, dipped in nitric acid, is then passed into the cavity, is made to pass over the epithelial lining surface, and is withdrawn. Usually, in its passage into the cavity of the tumor, it cauterizes sufficiently the lips of the incision to prevent any oozing of blood when the clamp forceps is removed. When the cyst is formed by the closure of a Meibomian duct, the better plan is to evert the lid and make the puncture from the conjunctival surface, the caustic being applied as directed. The advantage gained by this modification is in the more certain, thorough, and yet restricted application of the caustic, confining its cauterizing influences only to those portions in which action is desired. The results are in every case satisfactory. No after-treatment is needed.

